# High-flow nasal cannula in nonlaser microlaryngoscopic surgery: a prospective study of 19 cases in a chinese population

**DOI:** 10.1186/s12871-022-01627-3

**Published:** 2022-03-26

**Authors:** Bo Ma, Fei Liu, Dandan Wang, Ruihan Zhong, Kaihao Lin, Shuo Li, Jie Zhang, Chaoyang Li

**Affiliations:** 1 Department of Anesthesiology, Huazhong University of Science and Technology Union Shenzhen Hospital, Shenzhen, People’s Republic of China; 2 Department of Otorhinolaryngology, Huazhong University of Science and Technology Union Shenzhen Hospital, Shenzhen, People’s Republic of China

**Keywords:** High-flow nasal cannula, Transnasal humidified rapid-insufflation ventilatory exchange, Microlaryngoscopic surgery, Airway management, Apneic oxygenation

## Abstract

**Background:**

High-flow nasal cannula (HFNC) is a new type of oxygen therapy, but its application in surgery remains unclear, we tried to describe the application of HFNC in microlaryngoscopic surgery for the Chinese population.

**Methods:**

Nineteen adults, American society of anesthesiology class (ASA) 1–2 patients with body mass index < 30 kg.m^−2^ underwent microlaryngoscopic surgery using HFNC for airway management. Outcomes included apnoea time, intraoperative oxygenation, carbon dioxide value, lactate value, and the relationship between the duration of apnoea time and carbon dioxide levels.

**Results:**

A total of 19 patients underwent vocal cord tumor resection under a microlaryngoscope with HFNC as the sole method of ventilation. The mean age was 39.7 years old, and the mean BMI was 23.9 kg.m^−2^. The mean apnea time was 21.5 min. The SpO_2_ of 18 patients remained above 90%, and only 1 patient dropped to 88%. The average basal lactate and highest lactate value was 0.58 mmol. L^−1^ and 0.68 mmol.L^−1^. The difference between basal and highest lactate values was statistically significant (*P* < 0.05). The average highest PaCO_2_ value was 79.4 mmHg. The PaCO_2_ increased by 1.68 ± 0.12 mmHg every minute linearly.

**Conclusions:**

In the case series we have observed that HFNC would be safe and effective oxygenation and ventilation technique for selected Chinese patients undergoing non-laser microlaryngoscopic surgery within 30 min. The tubeless technology reduces the complications of tracheal intubation and jet ventilation and clears the surgical field of vision.

**Trial registration:**

Chinese Clinical Trial Registry (ChiCTR100049144).

## Background

Microlaryngoscopic surgery is widely used to treat a variety of oropharyngeal and laryngeal diseases. For the operation, the traditional way of oxygen supply is endotracheal intubation for mechanically controlled ventilation. However, the endotracheal tube occupies the surgical airway and blocks the surgical field, which limits the operation of surgeons. High-frequency jet ventilation can optimize the surgical field, but it can also cause barotrauma, tracheal mucosal edema, and ischemia. Recently, some groups have successfully used high-flow nasal cannula for oxygenation in microlaryngoscopic surgery. It could optimize the surgical field of vision and reduce endotracheal injury caused by tracheal intubation and air pressure injury caused by jet ventilation [1, 2]. 

A high-flow nasal cannula (HFNC) is a new type of oxygen therapy, which is a safe and effective therapy for the treatment of respiratory failure. It effectively heats and humidifies the outside air to 37℃, 100% relative humidity through a large-caliber soft nasal catheter. It delivers up to 70 L/min of airflow, and the inhaled oxygen concentration is from 21 to 100%. It is commonly applied in intensive care units and respiratory medicine [3]. During the induction and recovery phase of anesthesia, it is also commonly used to improve the oxygenation of patients, especially for patients with difficult airways [4].

In our case series, HFNC technology was used in patients with apnoea. The underlying mechanism of gas exchange is related to apneic oxygenation. In 1959, Frumin et al. [5] first reported the study on the principle of apneic oxygenation. The gas exchange in the alveoli is determined by the difference in gas concentration on both sides of the alveolar membrane and the solubility of different gases in the blood. The solubility of carbon dioxide in the blood is 25 times that of oxygen. The amount of carbon dioxide discharged into the alveoli during suffocation is only 10 ml.min^−1^, while the consumption of oxygen is 250 ml.min^−1^. Therefore, a negative pressure difference (− 240 ml.min^−1^) is generated in the alveoli. The negative pressure moves the gas in the oropharynx to the alveoli, which is the phenomenon of apneic oxygenation.

At present, the reports about the application of HFNC in laryngeal microsurgery is limited to a small number of case reports and retrospective analysis. In a case report, it was shown that HFNC oxygenation was used in laryngeal microsurgery, so the patients completed the operation, and there were no complications during the perioperative period [6]. In China, there is no corresponding report on the application of HFNC in laryngeal microsurgery. To optimize the surgical field of vision and reduce complications, we applied HFNC technology in microlaryngoscopic surgery for the first time to observe and evaluate the safety and feasibility of its application and tried to provide more evidence about the application of HFNC in laryngeal microsurgery for the Chinese population.

## Methods

The study was prospectively registered in the Chinese Clinical Trial Registry, the registration number is ChiCTR100049144, and the study was conducted at the Huazhong University of Science and Technology Union Shenzhen Hospital from December 2020 to August 2021.

### Study object

With the approval of the review committee of the Huazhong University of Science and Technology Union Shenzhen Hospital and the patient's signed informed consent, we conducted this prospective study. We selected 19 patients, all the patients met the followed clinical diagnostic criteria: range 18 to 65 years old, American society of anesthesiologists (ASA) physical status 1 to 2, mallampati grade 1–2, presenting for elective nonlaser microlaryngoscopic surgery lasting within 1 h. we excluded the patients with the followed condition: mouth opening less than 4.5 cm, the nail interval was less than 6.5 cm, the Body Mass Index (BMI) was greater than 30 kg.m^−2^, or the patients with lung diseases such as ( pneumonia, COPD, asthma, pulmonary hypertension, long-term smoking history), hypertension, or cerebrovascular disease.

### Study protocol

**Fig. 1:**
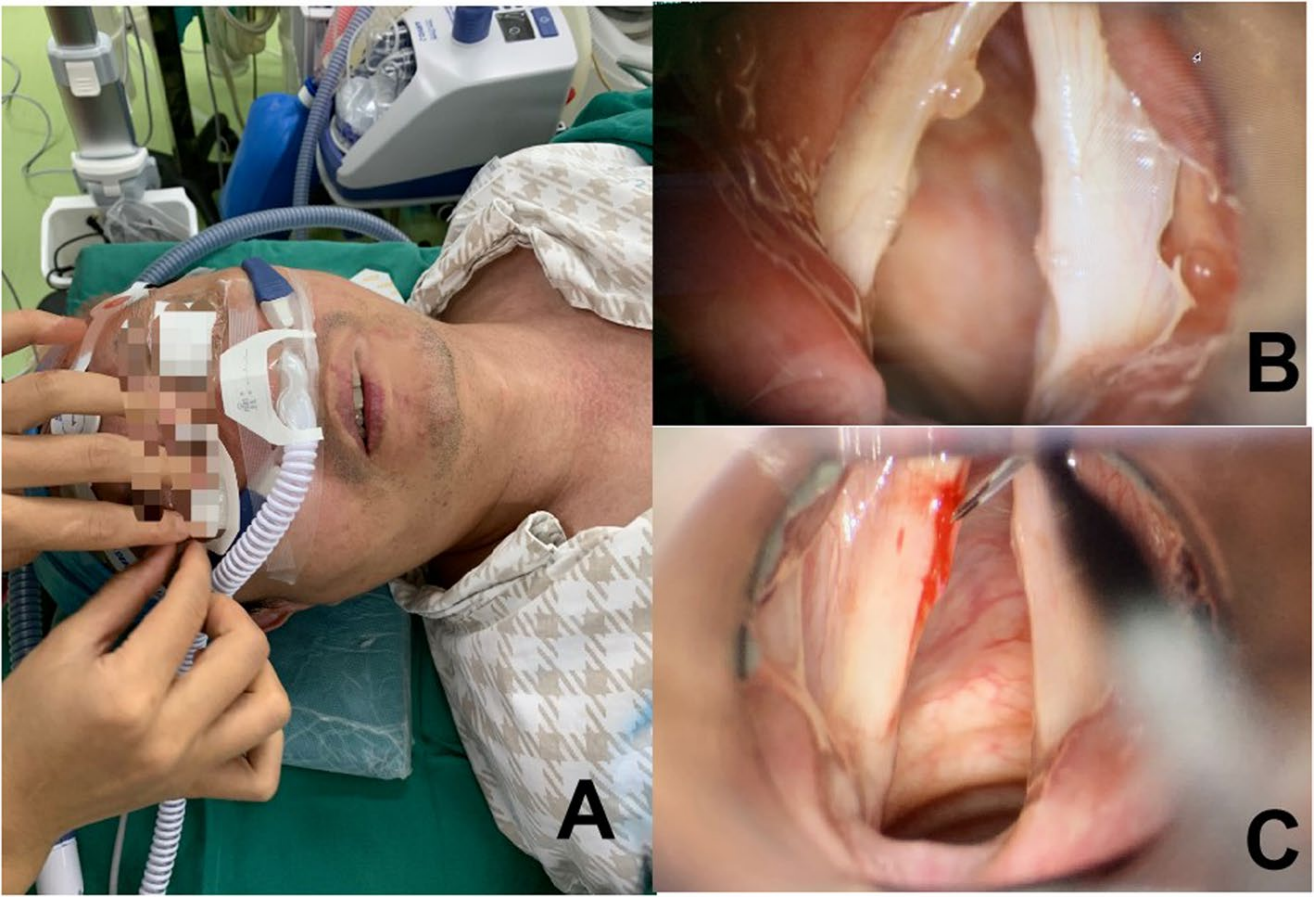
Intraoperative conditions of the application of HFNC. **A** Using HFNC as the ventilatory technique under general anesthesia. HFNC was administered using an Optiflow system (Fischer & Paykel Healthcare, Auck land, New Zealand), which delivered 37℃, 100% humidity, a FiO2 of 95% oxygen through a nasal cannula at 60 L/min. **B** The left vocal cord cyst could be seen clearly in the microlaryngoscope intraoperatively by using HFNC as the ventilatory technique. **C** Excision of the left Vocal Cord Cyst using HFNC with the adequate field of view intraoperatively. HFNC = High-flow nasal cannula

Preoperative and intraoperative monitoring included electrocardiogram, pulse oximetry, non-invasive blood pressure, arterial partial pressure of carbon dioxide (PaCO2), lactic acid, and potential of hydrogen( PH). Before induction of anesthesia, all the patients were pre-oxygenated with HFNC delivering 37℃, 100% humidity, a FiO2 of 95% oxygen through a nasal cannula at 60 L/min(Fig. 1A). All cases were anesthetized by using a total intravenous anesthetic technique with target-controlled infusions of propofol and remifentanil and using methylprednisolone 40 mg. Anesthesia was maintained with propofol and remifentanil with bispectral index monitoring. Succinylcholine was administered 1 mg/kg before inserting the supporting laryngoscope, which was considered the start of apnoea time. During the operation, HFNC set the same parameters as the pre-oxygenated process and was used for airway management. If the patient’s pulse oxygen saturation (SpO_2_) was lower than 90% consistently or the PaCO_2_ was greater than 110 mmHg, we immediately terminated the study and performed emergency airway management. Upon completion of the surgery, the mask was used to assist ventilation to eliminate carbon dioxide until the PaCO_2_ was below 60 mmHg. At the same time, HFNC was discontinued, the endpoint of apnoea time was recorded. Following by cessation of anesthetic infusion, the patients regained consciousness and were sent to the post-anesthesia care unit.

### Statistical analysis

SPSS16.0 software was used for data analysis. The measurement data were expressed as mean ± standard deviation or median (interquartile range). Normally the paired-sample t-test was used to compare the difference between before and after measurement data, and *P* < 0.05 was considered as the difference was statistically significant. The data were presented as means (standard deviations) or numbers (%). The correlation between the data of PaCO_2_ and time adopted one-variable linear correlation and regression analysis.

## Results

**Fig. 2:**
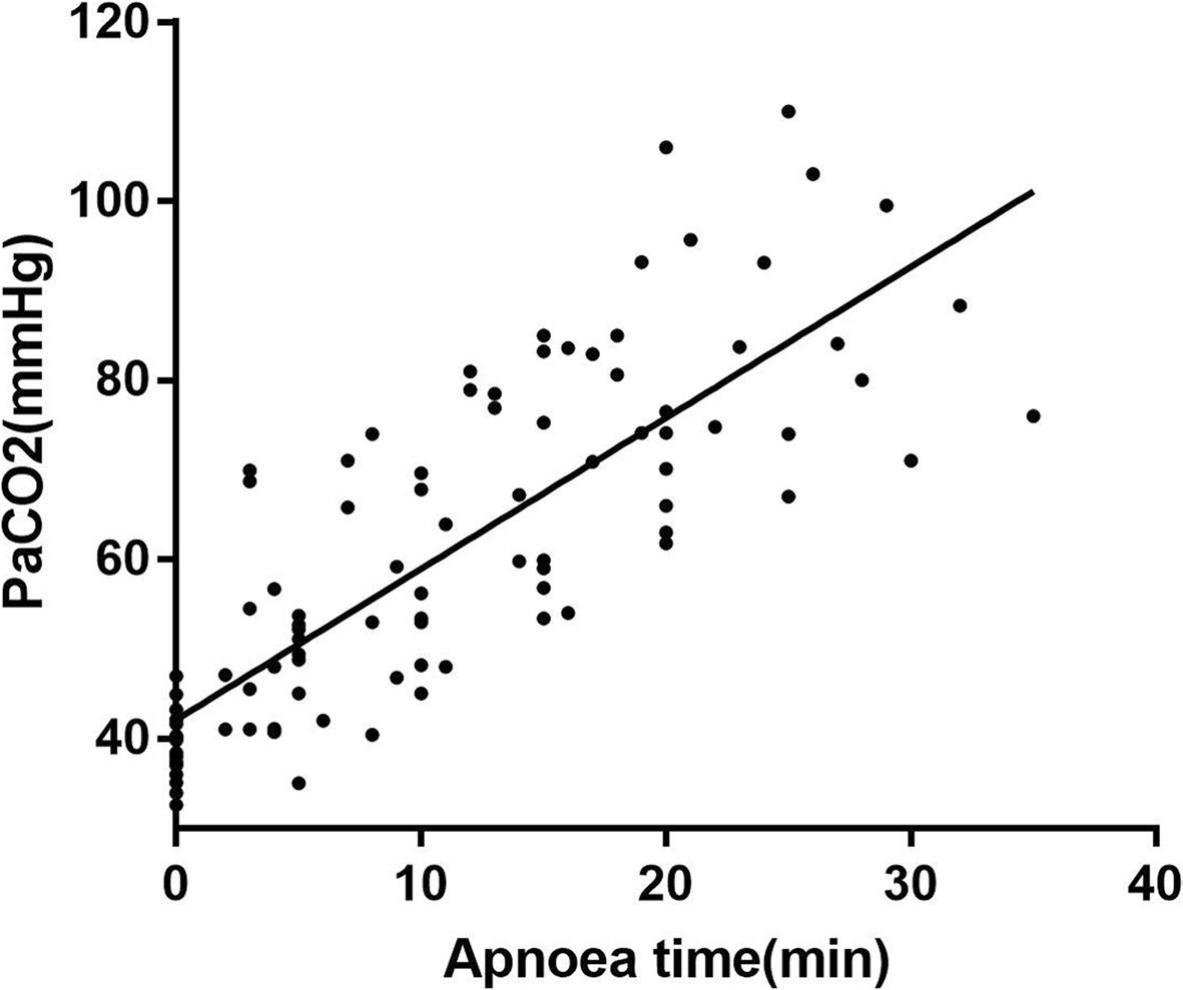
The relationship between apnoea time and PaCO_2_ levels. The line represents linear regression with *r* = 0.808 and *p* < 0.001. The regression equation was PaCO2 = (42.15 ± 1.78) + (1.68 ± 0.12) × apnoea time

This study comprised 19 patients, 8 male, and 11female, with a mean (SD) age of 39.7 (10.7) years old. The mean (SD) BMI was 23.9 (2.8) kg.m^−2^. The median (IQR) ASA grade was 1 (0,1), The median (IQR) mallampati grade was 1 (1,2) and the direct laryngoscopy grade was 1 (1.2). The mean (SD) apnoea time was 21.5 (6.6) min. The patients underwent vocal cord tumor resection under a microlaryngoscope. During the operation, the SpO_2_ of 18 patients remained above 90%, and the SpO_2_ of 1 patient dropped to 88% at the beginning of the operation, lasting about 1 min. Until the supporting laryngoscope was placed, the SpO_2_ rose to more than 90% after opening the airway. The average basal lactate value (SD) of all patients was 0.58 (0.19) mmol.L^−1^, and the average highest lactate value (SD) was 0.68 (0.29) mmol.L^−1^. The difference between basal and highest lactate values was statistically significant (*P* < 0.05). The average lowest PH value (SD) was 7.153 (0.06). The average basal PaCO_2_ value (SD) of the patients was 38.9 (3.8) mmHg, and the average highest PaCO_2_ value (SD) was 79.4 (15.2) mmHg (Table 1). PaCO_2_ increased by 1.68 ± 0.12 mmHg every minute linearly. Figure 2 illustrated the relationship between the duration of apnoea time and PaCO_2_ levels.

**Table 1:** Patient characteristics, apnoea time, SpO2, PaCO2, lactic acid, PH

Patient number	Age(years)	Sex	BMI (Kg.m^−2^)	ASA status	Apnoea time(min)	Lowest SpO2(%)	Baseline PaCO_2_(mmHg)	Highest PaCO_2_(mmHg)	Baseline Lac(mmol.L^−1^)	Highest Lac(mmol.L^−1^)	Lowest PH
1	41	F	20.8	1	20	97	38.0	74.1	0.6	0.6	7.156
2	43	F	29.2	1	20	88	36.0	76.5	0.7	0.8	7.161
3	65	F	26.0	2	14	94	33.9	59.8	0.4	0.5	7.204
4	23	M	23.8	2	28	99	35.9	80.0	0.8	1.1	7.197
5	49	F	24.6	1	25	94	37.4	110.0	0.3	0.5	7.040
6	29	F	22.3	2	25	100	35.1	74.0	0.6	0.6	7.152
7	36	F	21.0	1	15	100	32.6	75.3	0.5	0.5	7.124
8	23	M	20.9	1	19	98	44.9	93.2	0.6	0.7	7.130
9	50	M	22.8	2	19	97	42.1	74.1	0.9	0.9	7.153
10	36	F	25.4	2	10	99	39.8	54.5	0.6	0.8	7.296
11	53	F	23.5	1	23	91	40.1	83.7	0.2	0.2	7.143
12	33	F	23.9	2	29	98	38.4	99.5	0.5	0.5	7.067
13	48	M	23.2	2	26	95	41.6	103.0	0.7	0.7	7.072
14	29	M	28.4	1	17	95	43.2	82.9	0.7	1.4	7.165
15	37	M	20.7	1	15	99	40.3	83.2	0.6	0.6	7.172
16	33	M	27.8	2	32	97	47.0	88.3	0.9	1.1	7.134
17	48	F	22.0	1	20	99	36.0	66.0	0.5	0.5	7.178
18	38	F	20.7	1	16	100	37.0	54.0	0.3	0.3	7.215
19	41	M	27.9	2	35	97	40.0	76.0	0.7	0.7	7.149
Mean/Median	39.7		23.9	1	21.6	96.6	38.9	79.4	0.58	0.68	7.153
SD/IQR	10.7		2.8	0,1	6.6	3.2	3.8	15.2	0.19	0.29	0.06

*F* female, *M* male, *BMI* body mass index, *ASA* American standards association, *SpO2* peripheral oxygen saturation, *PaCO*_*2*_ arterial partial pressure of carbon dioxide, *PaO*_*2*_ arterial partial pressure of oxygen, *Lac* lactic acid, *PH* the potential of hydrogen, *SD* standard deviation, *IQR* interquartile range

## Discussion

HFNC is currently used for the respiratory treatment of various lung diseases and provides respiratory support for patients with spontaneous breathing and can also be used in operative rooms in various settings: during the preoxygenation before intubation or during apneic oxygenation without intubation. HFNC has been successfully used to provide ventilation and prolong apneic oxygenation without invasive airway in pediatric patients undergoing micro direct laryngoscopy and bronchoscopy [7]. In a randomized controlled trial including 48 children [8], HFNC exchange prolongs the safe apnea time in children. Another observational prospective study [4] assessed the apnea time in 25 patients with difficult planned intubation and undergoing general anesthesia. HFNC was administered continuously. The median apnea time was 14 (5–65) minutes, and no arterial oxygen desaturation was observed during apnea.

The underlying mechanism of using HFNC during microlaryngoscopic surgery is related to apneic oxygenation In the case series [5]. A physiological study using apnea oxygenation for laryngeal surgery has shown that this technique is suitable for patients with mild systemic diseases, the BMI is less than 30 kg.m^−2^, and the operation time does not exceed 30 min. The patients' SpO_2_ has never been lower than 91%. After using HFNC, it is expected to extend the apnea time, but in this process, carbon dioxide level monitoring is required [9].

Our study reveals that it is feasible to apply HFNC in microlaryngoscopic surgery. Compared with traditional airway management techniques, the application of HFNC in microlaryngoscopic surgery has several advantages. It does not occupy the surgical airway, which allows the surgeons to obtain a good surgical field of vision during the operation (Fig. 1B, C). To increase the safety of the study, we selected the patient population very strictly. The selected population was ASA1-2 and their BMI was less than 30 kg.m^−2^. Since high BMI is related to low functional residual capacity, it would be one of the most patient characteristics when using HFNC. Huang et al. found that patients with BMI > 30 kg.m^−2^ or bodyweight > 80 kg were 5 times more likely to require emergency ventilation than other patients [10]. Additionally, in a retrospective study including patients with HFNC, 13 patients had BMI > 30 kg.m^−2^, 4 of which required emergency tracheal intubation [11] . In our case series, the average asphyxia time was 21.6 min, of which the longest asphyxia time was 35 min. In 18 of the 19 patients, The SpO_2_ remained above 91% during this process. Only one patient whose BMI was 29.2 kg.m^−2^ had transient hypoxemia, which may be caused by airway obstruction. After placing a supporting laryngoscope, the oxygen saturation increased to a normal level which might be due to the opening of the airway. Therefore, for this case, we did not carry out emergency airway interventions such as tracheal intubation.

Although HFNC can extend the apnea time to a certain extent, hypercapnia and acidemia caused by carbon dioxide accumulation are the key limiting factors [12]. In a study, it was found that when HFNC was used for ventilation, the apnea time could be as long as 65 min, the SpO_2_ could be maintained at 90% or more, but the end-tidal carbon dioxide level reached up to 112 mmHg. Therefore, the operation time in our study was limited to less than 1 h. The highest value of PaCO_2_ was 110 mmHg (Table 1). None of the 19 patients had complications such as arrhythmia and electrolyte imbalance. After the operation, the patients regained consciousness. There was no delayed emergence in all of them. We recorded the PaCO_2_ levels of the 19 patients at different time points of asphyxia. Then, we analyzed and found that the level of PaCO_2_ increased linearly with time. PaCO_2_ increased by 1.68 ± 0.12 mmHg every 1 min, while a study was shown the end-tidal CO_2_ accumulation rate under low flow oxygen was 3.2 to 4.2 mmHg.min^−1^^13^. The possible reason is that HFNC can continuously flush out CO_2_ by flushing the dead space of the upper and lower airways and increasing the emission of carbon dioxide [14, 15].

The use of HFNC in microlaryngoscopic surgery has limitations. First of all, it is necessary to select suitable patients, which is not easy to popularize. Secondly, we should avoid using HFNC during prolonged surgery which will lead to the accumulation of carbon dioxide and produce a series of harmful physiological changes. Thirdly, since our project was the first time to observe HFNC’s application in microlaryngoscopic surgery for the Chinese population, safety was our primary concern, so we included a limited number of people to conduct the prospective observational study, but no randomized controlled trial. Once we have confirmed the safety of HFNC in surgery, we will expand the population sample and collect more information to conduct randomized controlled trials. Fourthly, our study lack assessment of surgeon feedback, which will be emphasized in our future studies. Lastly, there is a lack of data to confirm the safety of the technology in terms of airway fire risk. A case report showed that during a palate surgery, a monopolar diathermy grip was ignited when using HFNC [16]. For the sake of safety, the use of lasers in HFNC requires caution.

## Conclusion

In summary, in the case series, we have observed that HFNC would be safe and effective oxygenation and ventilation technique for selected Chinese patients undergoing non-laser microlaryngoscopic surgery within 30 min. The tubeless technology reduces the complications of tracheal intubation and jet ventilation and clears the surgical field of vision. In conclusions, we provide more evidence about the application of HFNC in laryngeal microsurgery for the Chinese population. 

## Data Availability

All data relevant to the study are included in the article or uploaded as supplementary information.
